# Impact of Additional Chromosomal Aberrations on the Disease Progression of Chronic Myelogenous Leukemia

**DOI:** 10.3389/fonc.2019.00088

**Published:** 2019-03-05

**Authors:** Ramachandran Krishna Chandran, Narayanan Geetha, Kunnathur Murugesan Sakthivel, Raveendran Suresh Kumar, Kumarapillai Mohanan Nair Jagathnath Krishna, Hariharan Sreedharan

**Affiliations:** ^1^Laboratory of Cytogenetics and Molecular Diagnostics, Division of Cancer Research, Regional Cancer Centre, Trivandrum, India; ^2^Division of Medical Oncology, Regional Cancer Centre, Trivandrum, India; ^3^Department of Biochemistry, PSG College of Arts and Science, Coimbatore, India; ^4^Division of Cancer Epidemiology and Biostatistics, Regional Cancer Centre, Trivandrum, India

**Keywords:** additional chromosomal aberrations, variant Ph translocation, Spectral Karyotyping, blast crisis, fluorescent *in situ* hybridization, Philadelphia Chromosome, chronic myeloid leukemia, GTG-banding

## Abstract

The emergence of additional chromosomal abnormalities (ACAs) in Philadelphia chromosome/*BCR-ABL1* positive chronic myeloid leukemia (CML), is considered to be a feature of disease evolution. However, their frequency of incidence, impact on prognosis and treatment response effect in CML is not conclusive. In the present study, we performed a chromosome analysis of 489 patients in different clinical stages of CML, using conventional GTG-banding, Fluorescent *in situ* Hybridization and Spectral Karyotyping. Among the *de novo* CP cases, ACAs were observed in 30 patients (10.20%) with lowest incidence, followed by IM resistant CP (16.66%) whereas in AP and BC, the occurrence of ACAs were higher, and was about 40.63 and 50.98%, respectively. The frequency of occurrence of ACAs were compared between the study groups and it was found that the incidence of ACAs was higher in BC compared to *de novo* and IM resistant CP cases. Likewise, it was higher in AP patients when compared between *de novo* and IM resistant CP cases, mirroring the fact of cytogenetic evolution with disease progression in CML. In addition, we observed 10 novel and 10 rare chromosomal aberrations among the study subjects. This study pinpoints the fact that the genome of advanced phase patients was highly unstable, and this environment of genomic instability is responsible for the high occurrence of ACAs. Treatment response analysis revealed that compared to initial phases, ACAs were associated with an adverse prognostic effect during the progressive stages of CML. This study further portrayed the cytogenetic mechanism of disease evolution in CML.

## Highlights

- Genomic instability in advanced phase CML patients is responsible for high occurrence of Additional Chromosomal aberrations (ACAs)
- Prognostic effect of ACAs in advanced phase CML were found to be adverse.

## Introduction

Chronic myelogenous leukemia (CML) is a hematopoietic disorder of multipotential stem cells, hallmarked by the cytogenetic event *t*(9;22)(q34;q11), and results in the generation of the Philadelphia (Ph) chromosome carrying *BCR-ABL1* fusion gene, which plays a central role in the pathogenesis of CML (1–3). Based on disease course and clinical characteristics, CML is often divided into the relatively indolent, early phase known as Chronic Phase (CP) and more aggressive advanced phase, consisting of an initial Accelerated Phase (AP) and a fatal Blast Crisis Phase (BC). Imatinib Mesylate (IM), a tyrosine kinase inhibitor, selectively binds and inhibits the tyrosine kinase activity of BCR-ABL1 oncoprotein revolutionizing the survival rate in CML. Survival rates are exceptionally high in CMLCP; however, therapy options for CML-AP and BC are very limited (4). This may be due to the biological complexity of the disease or the cascade of molecular events responsible for blastic transformation of CML, which remains inconclusive.

The emergence of additional chromosomal abnormalities (ACAs) and other associated genetic defects is considered a hallmark of multistep disease progression in CML. The accumulation of additional non-random cytogenetic aberrancies in Ph positive cells, known as “clonal evolution,” is considered to be the reflection of genetic instability that characterizes disease evolution in CML. Clonal evolution is normally allied with a decreased cytogenetic response to IM, increased risk of hematological relapse and a subsequent reduction in Overall Survival (5, 6). The frequency of ACAs is higher in CML-BC patients than in CML-CP or AP patients (7, 8). Additionally, CML-BC patients unveiled much more complex karyotypes in comparison with other phases of CML (9, 10).

Although it is well-known that chromosomal changes, besides Ph, are associated with evolution in CML, it is unclear if these cytogenetic changes are drivers of disease progression in CML and it needs to be elucidated if they do have any preference in the progressive environment of CML. Unraveling the cytogenetic anatomy of CML patients in different stages of CML is therefore extremely vital for proper treatment management of CML patients. In the present study, we therefore carried out the cytogenetic profiling of 489 patients in different clinical stages of CML using conventional (GTG-banding) and molecular cytogenetic techniques like FISH and SKY. Furthermore, to the best of our knowledge, the current study is one of the largest described sequences of cytogenetic examination in Indian CML patients.

## Results

### Cytogenetic Characteristics, Age, and Gender Distribution of Study Subjects

All study subjects tested positive for the *BCR- ABL1* fusion gene, as confirmed by FISH analysis. Among the study subjects, a successful cytogenetic analysis was obtained in 443 cases (90.59%) and in 46 cases (9.41%) the analysis could not be performed due to the poor morphology of the metaphases, or lack of cell division or failure of bone marrow culture. Of these 443 cases, ACAs were obtained in 80 cases. The study population consisted of 332 males and 157 females (M: F ratio = 2.1:1) with ages ranging from 15 to 75 years and the median age was 43 years.

### Cytogenetic Profile—Novel and Rare Chromosomal Aberrations Observed in Different Clinical Stages of CML

**Figure 1:**
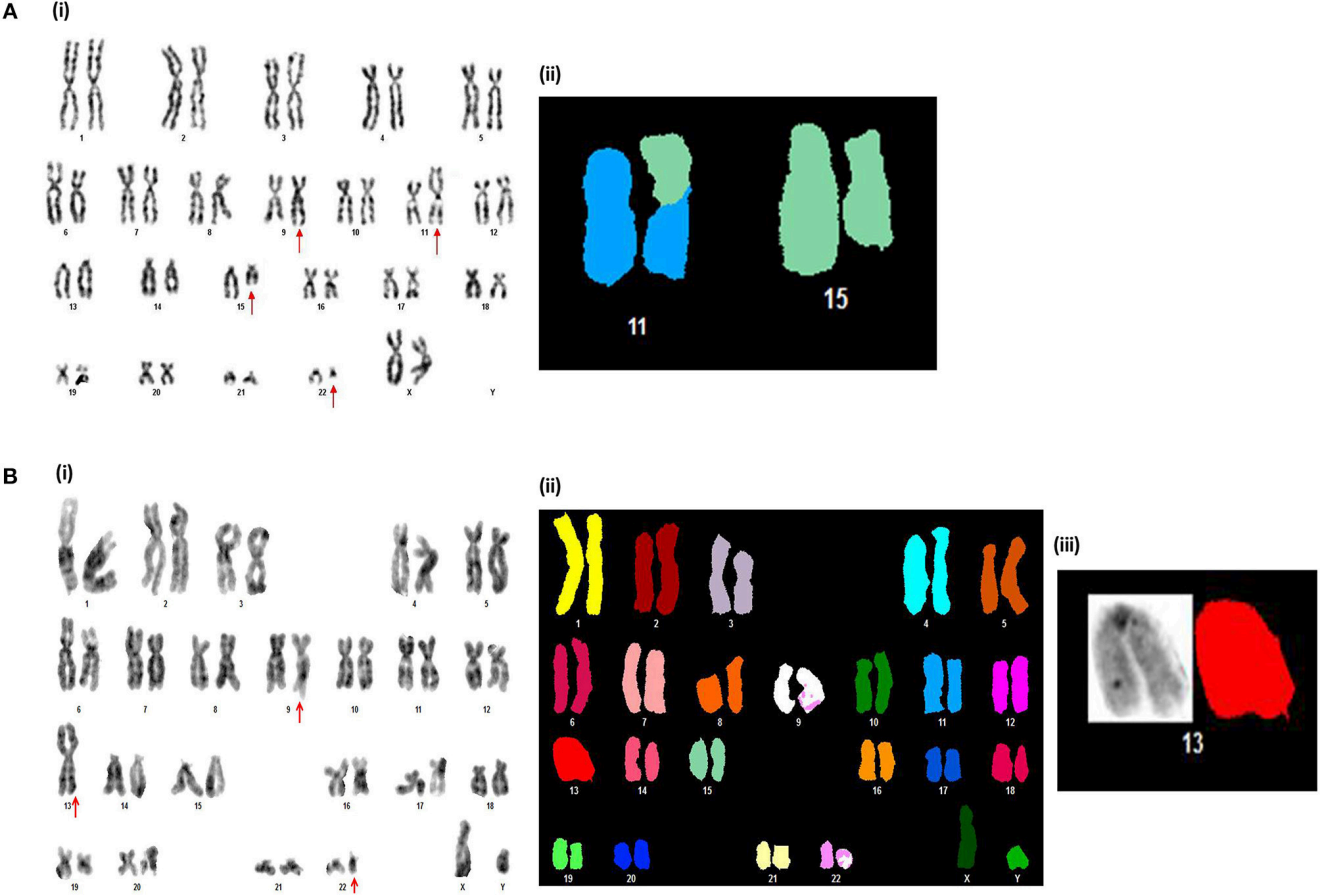
Novel chromosomal aberrations identified in *de novo* CP CML patients **(A)**. (i) G-banded karyotype showing 46,XX,*t*(9;22)(q34;q11),*t*(11;15)(p12;q15). (ii) Partial spectral karyotype confirming *t*(11;15)(p12;q15). **(B)** (i) G-banded karyotype showing 45,XY,*t*(9;22)(q34;q11),der(13;13)(q10;q10). (ii) Spectral karyotype, and (iii) Partial spectral karyotype confirming der(13;13)(q10;q10).

Out of 313 *de novo* CP patients, successful cytogenetic analysis was achieved in 294 cases and in 19 cases the analysis failed. Among the successfully analyzed cases, 264 patients (89.80%) carried the Ph chromosome as a sole cytogenetic abnormality while the remaining 30 patients (10.20%) carried ACAs (Table 1). Among these 30 cases, numerical aberrations were present in 14 cases (46.66%) whereas structural anomalies were seen in 15 cases (50%). One case possessed both structural and numerical aberrations simultaneously. Among the numerical aberrations, hyperdiploid (Moderate-hyperdiploidy with 2*n* = 47–50 and high-hyperdiloidy with 2*n* = 51–65) and polyploid (triploidy and tetraploidy) metaphases were most common. Among the structural aberrations, variant Ph translocations involving additional chromosomes other than chromosome 9 and 22, were most frequent. Moreover, molecular cytogenetic techniques like FISH and SKY along with GTG-banding revealed two novel and five rare chromosomal abnormalities in *de novo* CML-CP patients (Figure 1). The details of rare and novel anomalies identified in *de novo* CML-CP patients are depicted in Table 1.

**Table 1:** Cytogenetic profile of *de novo* CML-CP patients.

**Sl. No**	**Recurrent karyotype**	**No. of cases**
1	46,XY,*t*(9;22)(q34;q11)[20]	172
2	46,XX,*t*(9;22)(q34;q11)[20]	92
3	47,XX,*t*(9;22)(q34;q11),+der(22)*t*(9;22)[20]	1
4	47,XY,*t*(9;22)(q34;q11),+der(22)*t*(9;22)[10]/49,XY,+8,*t*(9;22)(q34;q11),+21,+der(22)*t*(9;22)[6]/49,XY,+8,*t*(9;22)(q34;q11),+16,+der(22)*t*(9;22)[4]	1
5	45,XY,-8,*t*(9;22)(q34;q11)[20]	1
6	46,XY,dup(5)(q35),del(8)(q23),*t*(9;22)(q34;q11)[20]	1
7	46,XY,*t*(9;22)(q34;q11),del(15)(q22)[20]	1
8	46,XY,*t*(9;22)(q34;q11),dup(15)(q26)[20]	1
9	46,XY,*t*(9;22)(q34;q11),dup(15)(p13)[20]	1
10	45,XY,*t*(9;22)(q34;q11),−20[12]/46,XY,*t*(9;22)(q34;q11)[8]	1
11	46,XX,*t*(9;22)(q34;q11)[11]/moderate hyperdiploidy, 2*n* = 47–50[9]	1
12	45,X,–Y,*t*(9;22)(q34;q11)[20]	2
13	46,XY,*t*(9;22)(q34;q11),dup(16)(q24)[20]	2
14	46,XY,*t*(9;22)(q34;q11)[10]/49,XY,+Y,+8,*t*(9;22)(q34;q11),+22[10]	1
15	46,XY,*t*(9;22)(q34;q11)[14]/High hyperdiploidy, 2*n* = 51–65[6]	2
16	46,XY,*t*(9;22)(q34;q11),dup(19)(q13.3)[20]	2
17	46,XX,*t*(9;22)(q34;q11)[15]/46,XX,*t*(9;22)(q34;q11),del(13)(q14)[5]	1
18	46,XX,*t*(9;22)(q34;q11)[13]**/**92 <4*n*>,XXYY[7]	1
19	46,XY,*t*(9;22)(q34;q11)[9]/47,XY,*t*(9;22)(q34;q11),+18[7]**/**92 <4*n*>,XXYY[4]	1
20	69 <3*n*>,XXX[20]	1
21	46,XX,*t*(9;22)(q34;q11)[10]/Moderate hypodiploidy, 2*n* = 40–45[10]	1
22	Karyotype failure	19
**RARE KARYOTYPE**		
23	46,XY,*t*(9;22;12)(q34;q11;q13)[20]	1
24	46,XX,*t*(9;22;13)(q34;q11;p11.1)[20]	1
25	46,XY,*t*(9;22;15)(q34;q11;q22)[20]	1
26	46,XX,*t*(9;22;16)(q34;q11;q24)[20]	1
27	46,XX,*t*(9;22)(q34;q11),*t*(15;15)(q22;q10)[20]	1
**NOVEL KARYOTYPE**		
28	46,XX,*t*(9;22)(q34;q11),*t*(11;15)(p12;q15)[20]	1
29	45,XY,*t*(9;22)(q34;q11),der(13;13)(q10;q10)[20]	1

**Figure 2:**
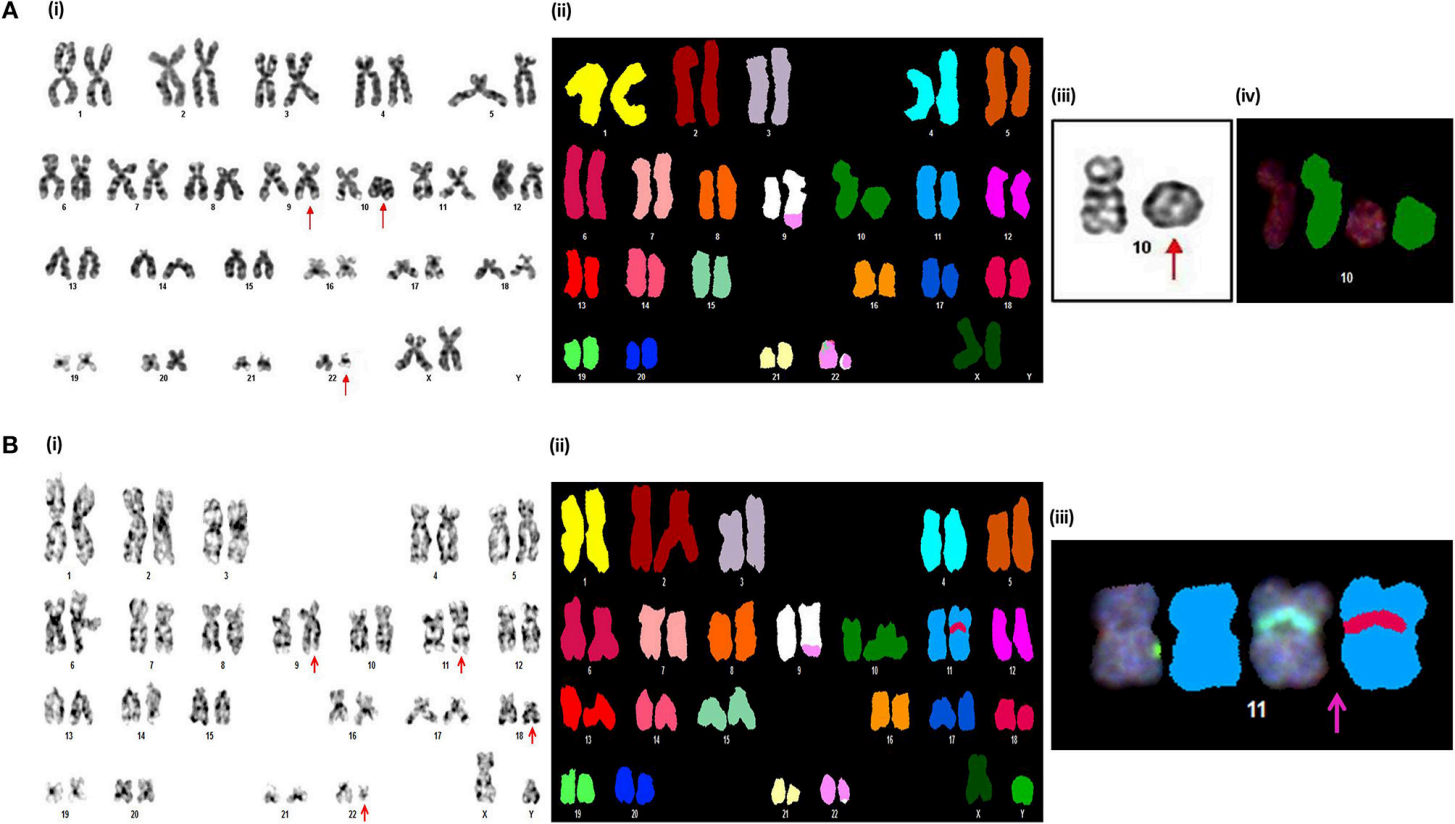
Novel chromosomal aberrations identified in CML AP patients **(A)** (i) G-banded karyotype showing 46,XX,t(9;22)(q34;q11),*r*(10)(p15q26). (ii) Spectral karyotype (iii) partial G-banded karyotype, and (iv) partial spectral karyotype confirming *r*(10)(p15q26). **(B)** (i) G-banded karyotype showing 46,XY,*t*(9;22)(q34;q11),ins(11;18)(p15;q21q23). (ii) Spectral karyotype (iii) partial spectral karyotype confirming ins(11;18)(p15;q21q23).

Of the 38 CML AP patients, cytogenetic analysis was successful in 32 patients. Among the 32 cases, 13 cases (40.63%) showed ACAs along with Ph. Of the 13 cases with ACAs, nine cases had numerical aberrations and four cases possessed structural anomalies (Table 2). Acquisition of an extra copy of the Ph chromosome was the main numerical anomaly associated with AP. Moderate hyperdiploid, tetraploidy, and trisomy 8 was also identified among numerical anomalies. A detailed cytogenetic analysis using the SKY technique revealed two novel chromosomal aberrations, 46,XX,*t*(9;22)(q34;q11),*r*(10)(p15q26) and 46,XY,*t*(9;22)(q34;q11),ins(11;18)(p15;q21q23) in this group (Figure 2). The details of novel anomalies identified in CML-AP patients are depicted in Table 2.

**Table 2:** Cytogenetic profile of CML-AP patients.

**Sl. No**	**Recurrent karyotype**	**No. of cases**
1	46,XY,*t*(9;22)(q34;q11)[20]	14
2	46,XX,*t*(9;22)(q34;q11)[20]	5
3	47,XX,*t*(9;22)(q34;q11),+der(22)*t*(9;22)[20]	2
4	47,XY,*t*(9;22)(q34;q11),+der(22)*t*(9;22)[20]	1
5	46,XY,*t*(9;22)(q34;q11)[15]/47,XY,*t*(9;22)(q34;q11),+der(22)*t*(9;22)[5]	1
6	46,XY,*t*(9;22)(q34;q11)[17]/47,XY,+8,*t*(9;22)(q34;q11)[3]	1
7	46,XX,*t*(9;22)(q34;q11),dup(17)(q24)[20]	1
8	46,XX,*t*(9;22)(q34;q11)[12]/49,XX,+9,*t*(9;22)(q34;q11),+21,+22[8]	1
9	46,XX,*t*(9;22)(q34;q11),dup(16)(q24)[20]	1
10	47,XX,*t*(9;22)(q34;q11),+13[13]/46,XX,*t*(9;22)(q34;q11)[7]	1
11	Moderate hyperdiploidy, 2*n* = 47–50[20]	1
12	46,XY,*t*(9;22)(q34;q11)[14]/92 <4*n*>,XXYY[6]	1
13	Karyotype failure	6
**NOVEL KARYOTYPE**		
14	46,XX,*t*(9;22)(q34;q11),*r*(10)(p15q26)[20]	1
15	46,XY,*t*(9;22)(q34;q11),ins(11;18)(p15;q21q23)[20]	1

**Figure 3:**
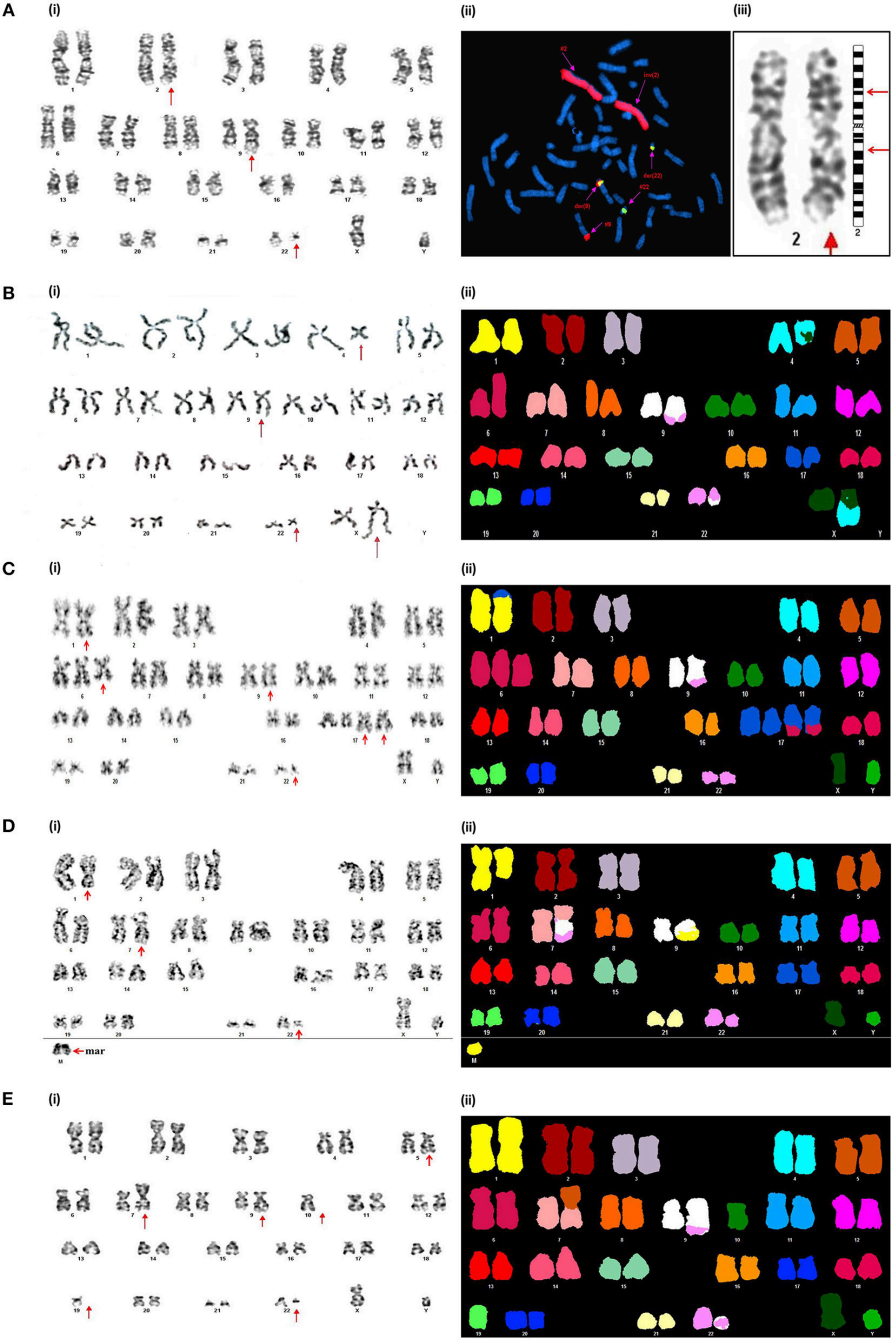
Novel chromosomal aberrations identified in CML BC patients **(A)** (i) G-banded karyotype showing 46,XY,inv(2)(p14q21),*t*(9;22)(q34;q11). (ii) Metaphase FISH using WCP-2 along with *BCR-ABL1* DCDF translocation probe confirmed the absence of genetic material exchange of chromosome 2 with other chromosomes. (iii) Partial G-banded karyotype showing pericentric inversion of chromosome 2. **(B)** (i) G-banded karyotype showing 46,X,t(X;4)(q21;q34),t(9;22)(q34;q11). (ii) Spectral karyotype confirming *t*(X;4)(q21;q34). **(C)** (i) G-banded karyotype showing 49,XY,der(1)*t*(1;17)(p36.3;q25),+6,*t*(9;22)(q34;q11),+der(17)*t*(6;17)(q22;q25)×2. (ii) Confirmation of the complex karyotype by using Spectral karyotyping. **(D)** (i) G-banded karyotype showing 47,XY,der(7)*t*(7;9)(p11.2;q11)*t*(9;22) (q34;q11),der(9)*t*(1;9)(q32;q11),+mar. (ii) Confirmation of the complex karyotype by using Spectral karyotyping. **(E)**. (i) G-banded karyotype showing 44,XY,der(7)*t*(5;7)(q21;p11.2),*t*(9;22)(q34;q11),-10,-19. (ii) Confirmation of the complex karyotype by using Spectral karyotyping.

While in the BC phase of CML, conventional cytogenetic analysis unmasked ACAs in 26 patients (50.98%) out of the 51 cases that could be analyzed, and the remaining patient possessed a karyotype with *t*(9;22)as a sole cytogenetic anomaly. Of the 26 cases with ACAs, numerical chromosomal changes were observed in 15 cases, and 11 patients had structural chromosomal rearrangements (Table 3). Here, the occurrence of multiple copies of the Ph (7/15 cases) was the main route of numerical associated clonal evolution. The frequency of incidence of hyperdiploid (moderate and high hyperdiploidy) and polyploid (both triploidy and tetraploidy) metaphases were also high in this phase. Trisomy 8, loss of the Y chromosome, low hypodiploidy with 2*n* = 30–39 and monosomy 17, were also identified in numerical abnormal cases. Since the genome of BC-CML patients harbors far more chromosomal rearrangements, due to clonal events, we combined the classical GTG-banding technique with the FISH and SKY techniques to unravel the spectrum of complex aberrations. In the spectrum of anomalies, five novel and three rare chromosomal rearrangements were identified (Figure 3). The novel and rare karyotypes identified in CML-BC patients are illustrated in Table 3.

**Table 3:** Cytogenetic profile of CML-BC patients.

**Sl. No**	**Recurrent karyotype**	**No. of cases**
1	46,XY,*t*(9;22)(q34;q11)[20]	21
2	46,XX,*t*(9;22)(q34;q11)[20]	4
3	47,XX,*t*(9;22)(q34;q11),+der(22)*t*(9;22)[20]	1
4	47,XX,*t*(9;22)(q34;q11),+der(22)*t*(9;22)[10]/46,XX,*t*(9;22)(q34;q11)[10]	1
5	47,XY,*t*(9;22)(q34;q11),+der(22)*t*(9;22)[20]	2
6	47,XY,*t*(9;22)(q34;q11),+der(22)*t*(9;22)[16]/46,XY,*t*(9;22)(q34;q11)[4]	1
7	48,XY,*t*(3;21)(q26;q22),*t*(9;22)(q34;q11),+der(22)t(9;22),+12[20]	1
8	47,XY,+8,*t*(9;22)(q34;q11)[20]	2
9	46,XX,*t*(9;22)(q34;q11)[12]/45,XX,*t*(9;22)(q34;q11),−10[8]	1
10	49,XX,+9,*t*(9;22)(q34;q11),dup(16)(q24),+21,+der(22)*t*(9;22)[20]	1
11	Moderate hyperdiploidy, 2*n* = 47–50[20]	1
12	High hyperdiploidy, 2*n* = 51–65[20]	1
13	Moderate hyperdiploidy, 2*n* = 47–50[11]/92 <4*n*>,XXYY[9]	1
14	High hyperdiploidy, 2*n* = 51–65[20]	1
15	46,XX,*t*(9;22)(q34;q11)[15]/High hyperdiploidy[5]	1
16	46,XY,*t*(9;22)(q34;q11)[9]/48,XY,*t*(9;22)(q34;q11),+18,+der(22)*t*(9;22) [7]/92 <4*n*>,XXYY[4]	1
17	46,XX, *t*(9;22)(q34;q11)[11]/69 <3*n*>XXX[9]	1
18	46,XY,*t*(9;22)(q34;q11)[11]69 <3*n*>XXY[9]	1
19	Karyotype Failure	9
**RARE KARYOTYPE**		
20	45,XX,der(7)*t*(7;8)(p?;q?),−8,*t*(9;22)(q34;q11)[20]	1
21	45,XX,−8,*t*(9;22;1)(q34;q11;q12)[20]	1
22	46,XX,*t*(9;22;6)(q34;q11;q25)[20]	1
**NOVEL KARYOTYPE**		
23	47,XY,der(7)*t*(7;9)(p11.2;q11)*t*(9;22)(q34;q11),der(9)*t*(1;9)(q32;q11),+mar[20]	1
24	46,XY,inv(2)(p14q21),*t*(9;22)(q34;q11)[20]	1
25	44,XY,der(7)*t*(5;7)(q21;p11.2),*t*(9;22)(q34;q11),−10,−19[20]	1
26	49,XY,der(1)*t*(1;17)(p36.3;q25),+6,*t*(9;22)(q34;q11),+der(17)*t*(6;17)(q22;q25) × 2[20]	1
27	46,X,*t*(X;4)(q21;q34),*t*(9;22)(q34;q11)[20]	1

**Figure 4:**
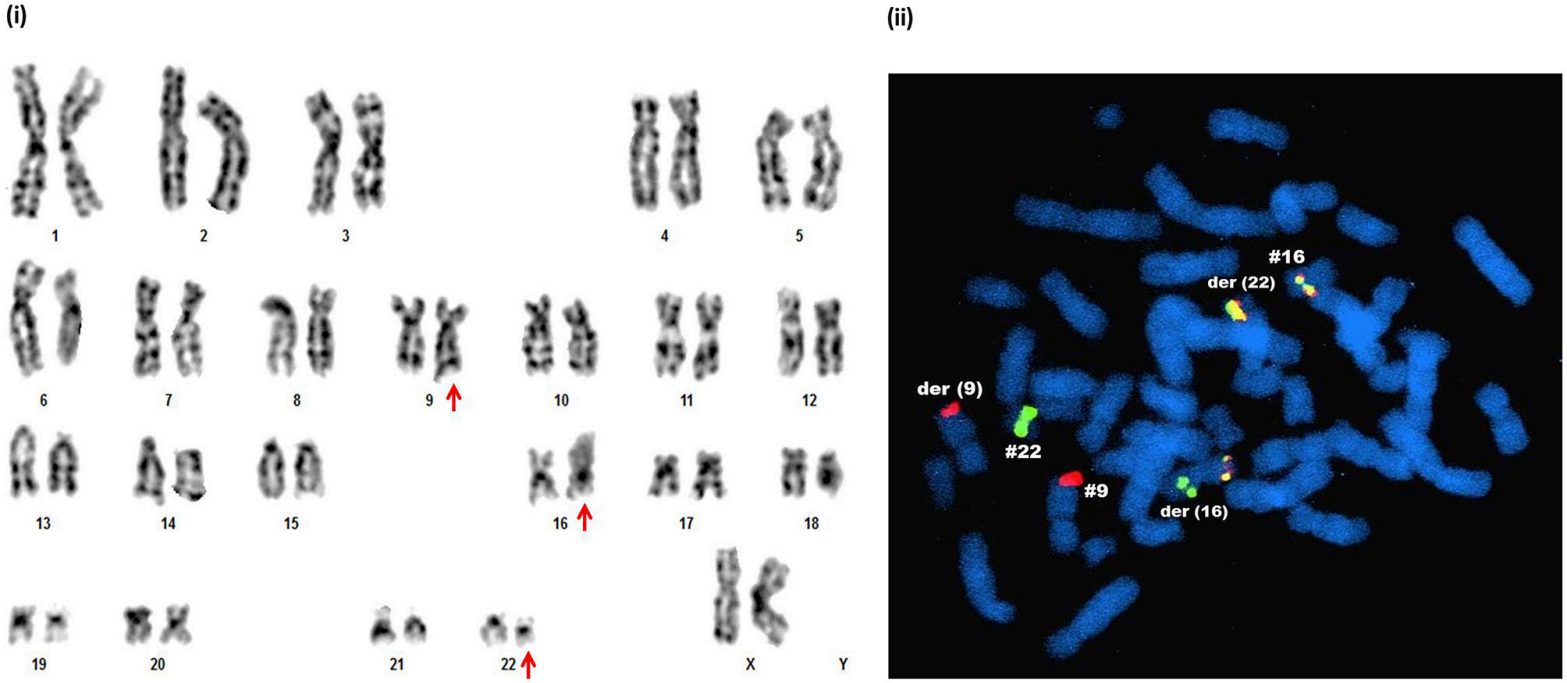
Novel chromosomal aberrations identified in IM resistant CML CP patients. (i) G-banded karyotype showing 46,XX,*t*(9;22;16)(q34;q11;p11.2). (ii) Metaphase FISH confirmation of *t*(9;22;16)(q34;q11;p11.2) by DCDF *BCR-ABL1* probe and *CBF*β*-MYH* break apart probe, which detected two red (*ABL1* genes on chr.9 and der chr.9), one green (*BCR* gene on chr.22), two red-yellow-green (*BCR-ABL1* fusion gene on der(22) and *CBF*β*-MYH* fusion gene on chr.16) signals. One red-yellow-green (*CBF*β*-MYH* fusion gene) and green (*BCR*) signals together in the chromosome indicated derived chromosome16.

With regards to the IM resistant CP patients, successful cytogenetic analysis was performed in 66 cases out of the 78 patients analyzed and 11 patients (16.66%) showed ACAs. The frequency of numerical aberrations here was also higher compared to structural aberrations (7/11 cases) (Table 4). Similar to AP and BC, the emergence of multiple copies of the Ph chromosome was the main culprit for numerical associated aberrations in this group. Moreover, two IM resistant patients revealed the presence of two to three copies of the isoderivative chromosome 22 [ider(22)] which resulted in the 2 to 6-fold amplification of the Ph chromosome. In addition, both metaphase FISH analysis and GTG-banding unraveled a novel Ph chromosome variant involving chromosome 16, along with chromosome 9 and 22 (Figure 4). The complex karyotypes of two rare cases and the complete karyotype of novel aberration are detailed in Table 4.

**Table 4:** Cytogenetic profile of IM Resistant CML-CP patients.

**Sl. No**	**Recurrent karyotype**	**No. of cases**
1	46,XY,*t*(9;22)(q34;q11)[20]	31
2	46,XX,*t*(9;22)(q34;q11)[20]	16
3	46,XY,*t*(9;22)(q34;q11)[10]/46,XY[10]	2
4	46,XY,*t*(9;22)(q34;q11)[14]/46,XY[6]	2
5	46,XY,*t*(9;22)(q34;q11)[16]/46,XY[4]	2
6	46,XX,*t*(9;22)(q34;q11)[12]/46,XX [8]	2
7	47,XY,*t*(9;22)(q34;q11),+der(22)*t*(9;22)[20]	1
8	47,XY,*t*(9;22)(q34;q11),+der(22)*t*(9;22)[10]/49,XY,+8,*t*(9;22)(q34;q11),+der(22)*t*(9;22),+21[7]/49,XY,*t*(9;22)(q34;q11),+der(22)*t*(9;22),+16,+21[3]	1
9	46,X,–Y,*t*(9;22)(q34;q11),+der(22)*t*(9;22)[11]/47,XY,*t*(9;22)(q34;q11), +der(22)*t*(9;22)[9]	1
10	45,X,–Y,*t*(9;22)(q34;q11)	2
11	47,XX,+8,*t*(9;22)(q34;q11)[20]	2
12	46,XX,*t*(9;22)(q34;q11),dup(16)(q24)[20]	1
13	Karyotype failure	12
**RARE KARYOTYPE**		
14	46,XY,der(9)*t*(9;22)(q34.13;q11.23),ider(22)(p12)*t*(9;22)[7]/47,sl,+ider(22)[21]/48,sdl,+ider(22)[2]	1
15	46,XY,*t*(9;22)(q34.13;q11.23)[4]/46,–der(22)*t*(9;22),+ider(22)(p12) *t*(9;22)[4]/47,sdl1,+ider(22)[13]/48,sl,+der(22),+ider(22)[4]/47,sl,+8[5]	1
**NOVEL KARYOTYPE**		
16	46,XX,*t*(9;22;16)(q34;q11;p11.2)[20]	1

### Comparison of Cytogenetic Data Between the Study Groups—Advanced Phase CML Patients Were More Vulnerable to Additional Chromosomal Rearrangements

**Figure 5:**
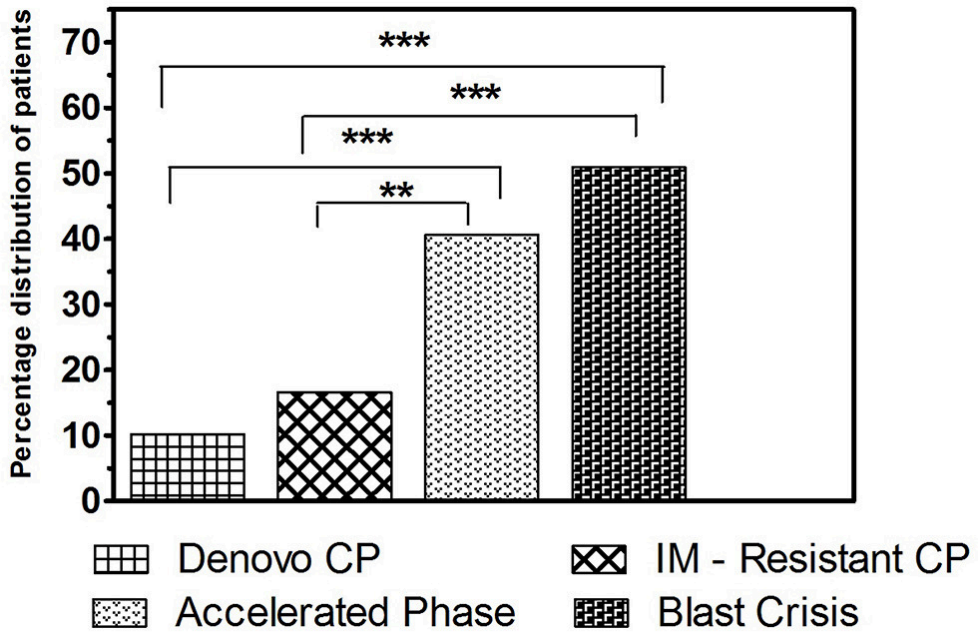
The frequency of incidence of ACAs among the study groups. Bar graph showing the frequency of incidence of additional chromosomal aberrations in different clinical stages of CML (***P < 0.001, **P < 0.01).

Cytogenetic findings observed in each clinical stages of CML were analyzed and compared, and it was found that the frequency of occurrence of ACAs were higher in advanced phases of the disease such as AP and BC, when compared to *de novo* and IM resistant CP patients. In a comparison of BC and *de novo* CP, BC showed a significantly higher frequency of ACAs (50.98% vs. 10.20%, *P* < 0.0001). Likewise, in comparing BC and IM resistant CP, BC displayed a significantly higher rate of incidence of ACAs (50.98% vs. 16.66%, *P* < 0.0001). The same analysis between AP and *de novo* CP, revealed that the rate of incidence of chromosomal changes other than *t*(9;22) were significantly higher in AP (40.63% vs. 10.20%, *P* < 0.0005). Compared to IM resistant CP, AP also showed a significantly higher rate of chromosomal rearrangements besides *t*(9;22) (40.63% vs. 16.66%, *P* < 0.009). No significant differences were observed in the occurrence of ACAs between AP and BC (*P* > 0.3) as well as in the *de novo* CP and IM resistant CP stage (*P* > 0.1) (Figure 5).

### Correlation of ACAs With Hematological Parameters

Hematologic parameters such as WBC count, platelet, Hb, BM blast, PB blast, and LDH levels were analyzed between patients with ACAs and patients with *t*(9;22) as the sole cytogenetic abnormality, in all the study stages of CML, in order to reveal any significant relationship. However, only the BC phase patients with ACAs showed significantly higher Hb levels compared to patients with *t*(9;22) alone (*P* = 0.017). In all other study groups, no significant relationship was observed between any of the hematological parameters and ACAs (Table 5).

**Table 5:** Comparison of clinical & laboratory characteristics between cases with Ph as sole cytogenetic aberration & cases with ACAs in CML patients.

**Clinical parameters**	**Karyotype**	***De novo*** **CP**		**IM Resistant CP**		**Accelerated phase**		**Blast crisis**	
		**Mean**	***P*-value**	**Mean**	***P*-value**	**Mean**	***P*-value**	**Mean**	***P*-value**
Hb, gm%	Ph alone	11.02	0.761	10.39	0.738	9.71	0.331	8.70	0.017
	ACAs	10.90		10.11		9.14		10.00	
WBC, × 10^9^/L	Ph alone	268.79	0.389	327.83	0.150	153.82	0.511	114.68	0.665
	ACAs	258.51		162.00		170.31		257.39	
PLC, × 10^9^/L	Ph alone	364.51	0.346	261.70	0.448	335.75	0.988	200.24	0.126
	ACAs	405.33		236.56		378.98		152.04	
BM Blast,%	Ph alone	4.56	0.202	2.51	0.150	12.58	0.141	48.92	0.144
	ACAs	4.35		4.11		14.21		59.61	
PB Blast,%	Ph alone	4.30	0.675	1.80	0.182	12.38	0.165	48.27	0.936
	ACAs	4.13		2.78		13.71		48.74	
LDH, IU/L	Ph alone	2457.36	0.630	2471.44	0.515	2593.55	0.799	3845.42	0.139
	ACAs	2648.00		2538.28		2725.56		2999.58	

*Hb, Hemoglobin; WBC, White Blood Cell Count; PLC, Platelet Count; BM, Bone Marrow; PB, Peripheral Blood; LDH, Lactate dehydrogenase; ACAs, Additional Chromosomal Aberrations; Ph, Philadelphia Chromosome. All statistical calculations were performed using SPSS software version 21. Student's t-test and Mann Whitney U-test was used to find the difference in the distribution of covariates such as Hb (gm%), TC (cmm), Platelet (cmm), LDH (IU/L), PB Blast (%), and BM blast (%) between cases with Ph as sole cytogenetic aberration and cases with ACAs. A P < 0.05 was taken as statistically significant*.

## Discussion

In 1956, Tjio and Levan discovered that 46 chromosomes were present in humans, after which many efforts followed to study the role of chromosomal abnormalities in human cancers (11). In 1960, Nowell and Hungerford discovered that a minute abnormal chromosome 22, called the Philadelphia Chromosome, remarked first time cancer with a specific genetic abnormality, which created a new era of genetic diagnosis (12). Thirteen years later in June 1973, with the advent of the new chromosomal banding techniques, Janet Rowley from Chicago revealed that this remarkable Philadelphia chromosome was formed as result of the balanced reciprocal translocation of genetic material between the long arms of chromosomes 9 and 22, *t*(9;22)(q34;q11) (13). Later works showed that BCR-ABL1 oncoprotein, with constitutive tyrosine kinase activity, was generated from this balanced translocation which leads to leukemogenesis in CML (14, 15).

Recent advancement in the area of genetic knowledge has shown a strong association between specific cytogenetic abnormalities, with the diagnosis and prognosis of certain kinds of neoplasms, thereby extending cancer cytogenetics into clinical practice from research laboratories. In India, cytogenetic studies in CML patients are underway with different chromosomal abnormalities being reported in different parts of the country (16–18). However, the data on the effect of cytogenetic mechanisms that cause transformation and disease progression to blast crisis of CML, still remains rather elusive in the Indian population; in spite of the large data accumulated worldwide in recent years. The current study, consisting of cytogenetic data from 489 CML cases, one of the largest series of CML patients in India, to the best of our knowledge, further portrays the cytogenetic nature on the disease progression of CML.

Out of 489 cases, 332 patients were male and 157 were female, with a male to female ratio of 2.1:1. The male dominance in our study group was in accordance with previous reports that males had a higher risk developing CML, or a shorter latency from initiation to diagnosis of CML (19). The age of our study samples ranged from 15 to 75 years, with a median age of 43 years, similar to previous reports (20, 21). Sixty five years in the UK (22), 65 years in the US (23), 60.3 years in Germany (22), and 55 years in France (24) with the youngest being reported in Asian populations (36–38 years in Thailand, 43 years in Singapore, and 37 years in South Korea) (21). The younger age of CML incidence in Asian population might be due to a higher exposure of air pollutants compared to western nations (25). Regional and ethnic variations may also play a role (26).

In the current study, conventional cytogenetic analysis through GTG-banding unveiled additional chromosomal abnormalities along with the classical Ph chromosome in all the study groups of CML, including IM resistant CML-CP. Among the *de novo* CP cases analyzed, ACAs were observed in 30 patients (10.20%) with lowest incidence, followed by the therapy resistant CP group (16.66%), whereas in AP and BC the occurrence of ACAs were higher at around 40.63 and 50.98%, respectively. These results were in accordance with previous studies and it was reported that ACAs were common in CML with a high incidence rate in AP and BC (27, 28). Furthermore, similar to our data, more studies reported that 5–10% of patients in CP, 30–40% of patients in AP and 50–80% of patients in BC showed ACAs in addition to the Ph chromosome (26, 29).

The frequency of occurrence of ACAs were compared between the study groups and it was found that the frequency of incidence of ACAs were significantly higher in BC compared to *de novo* and IM resistant CP groups (50.98% vs. 10.20%, 50.98% vs. 16.66%, all *P* < 0.0001). Likewise, it was significantly higher in AP patients when compared between *de novo* and IM resistant CP cases (40.63% vs. 10.20%, *P* < 0.0005 and 40.63% vs. 16.66%, *P* < 0.009, respectively). In the present study, it was obvious that there was a gradual increase in the occurrence of clonal evolution from the CP to BC stage of CML, revealing that the genomic instability in higher phases of CML might play a major role in the development of ACAs. Moreover, our study revealed that BC patients with ACAs had presented a higher Hb level than BC patients without ACAs. However, neither Hb levels nor total count contributed any relevant information regarding the prognostic effect in CML (30, 31).

Even though the association between ACAs and its effect on disease progression is known, very little is known about the role of each individual ACAs (32). However, many studies in CML have classified ACAs into major and minor routes of abnormalities (33–35). As previously described (28, 33, 36), acquisition of extra copies of Ph, trisomy 8, and trisomy 19 were the most frequent major route anomalies observed, while loss of Y chromosome, trisomy 21, hypodiploidy, hyperdiploidy, and polyploidy were the most frequent minor route aberrations observed among the study subjects. All five patients with loss of Y were younger which is contradictory to previous reports where loss of the Y chromosome was frequently associated with older age (37). Similar to a previous report (26), the current study revealed that the frequency of incidence of hyperdiploid and polyploidy metaphases were high in BC. In our study, two cases of IM resistance CP developed two to five copies of Ph in the form of the isoderivative chromosome (22), a rare cytogenetic event in CML, which formed as a result of the fusion of two Ph chromosomes at the satellite region of their short arms, through either fusion or translocation (38).

Although, a positive correlation between ACAs and disease evolution in CML exists, the effect of these secondary chromosomal rearrangements on disease prognosis is not conclusive. Previous studies suggested that ACA during AP/BC was associated with a worse prognostic effect (39). Similarly, the current study also unveiled that both major and minor route anomalies emerged during AP and BC, which was allied with a poor prognostic effect. However, these secondary chromosomal anomalies during CP at the time of initial diagnosis conferred a favorable outcome. This discrepancy in prognosis might be due to clonal evolution events. Consistent with our findings, it was implicated that ACAs that arose during the course of IM therapy in CML patients, was treated as a synonym of disease evolution and indicated an adverse impact on prognosis (6). Contradictory to our findings, previous studies (40, 41) showed that CML patients with major route ACAs at diagnosis took a longer time to achieve CCyR and MMR (Major Molecular Response) and possessed shorter PFS and OS compared to patients with standard *t*(9;22). In addition, they also proved that the major route ACAs at diagnosis was allied with a negative impact on survival and signified disease progression in CML. According to Majlis et al. (42) and Cortes et al. (43) the prognostic relevance of ACAs are not uniform, and depended on various factors like the specific anomaly, its frequency of analysis in metaphase, the time of occurrence, relation with other AP features and the mode of therapy in CML.

The other most common cytogenetic abnormality observed in our study group was variant Ph translocation, which involve additional partner chromosomal regions besides 9 and 22. The present study identified seven patients (1.43%) with variant Ph translocations, in agreement with a previous study that showed 2–10% of CML patients had complex variant Ph translocations (44). In our study, the occurrence of variant Ph translocations was high in CP patients, especially at the time of diagnosis, in agreement with a previous report (39). According to the Mitelman database (45), among the variant Ph translocations identified in our study, one was novel and six were rare in CML. The novel variant translocation, 46,XX,*t*(9;22;16)(q34;q11;p11.2), was observed in a 70-years-old female patient with IM resistant CP. In the present study, chromosomes 1, 6, 12, 13, 15, and 16 were performed as the third chromosome partner. Like previous observations, all the *de novo* CML-CP cases with variant Ph in our study population, achieved a complete cytogenetic response with IM therapy (46, 47). However, emergence of variant Ph during BC and IM resistant CP conferred a worse prognosis.

The contribution of rare and novel chromosomal rearrangements in CML is unclear and very few have been mapped in detail. The exact consequences of such rare and novel chromosomal aberrations identified in the present study need to be elucidated, however they could represent an important genetic event in the leukemogenesis, resistance to chemotherapeutic agents and disease evolution in CML. The present study characterized all such chromosomal rearrangements in detail and identified 10 rare and 10 novel chromosomal aberrations (45). The novel chromosomal changes observed in BC included three complex karyotypes [47,XY,der(7)*t*(7;9)(p11.2;q11)*t*(9;22)(q34;q11),der(9)*t*(1;9)(q32; q11),+mar; 49,XY,der(1)*t*(1;17)(p36.3;q25),+6,*t*(9;22)(q34;q11),+der(17)*t*(6;17)(q22;q25) × 2[20]; 44,XY,der(7)*t*(5;7)(q21;p11.2),*t*(9;22)(q34;q11),−10,−19] and two other chromosomal rearrangements such as 46,XY,inv(2)(p14q21),*t*(9;22)(q34;q11) and 46,X,*t*(X;4)(q21;q34),*t*(9;22)(q34;q11). All of these novel aberrations identified in CML-BC were associated with an adverse impact on prognosis.

Among the novel anomalies identified in the *de novo* CP [46,XX,*t*(9;22)(q34;q11),*t*(11;15)(p12;q15) and 45,XY,*t*(9;22)(q34;q11),der(13;13)(q10;q10)], cases with *t*(11;15)(p12;q15) achieved complete cytogenetic and molecular remission by 12 months with IM therapy. However, the other patient with Robertsonian translocation or der(13;13)(q10;q10) showed a minimal cytogenetic response. It was reported that this type of Robertsonian translocation involving the homologous chromosomes is very rare in human cancers (45). 46,XX,*t*(9;22)(q34;q11),*r*(10)(p15q26) and 46,XY,*t*(9;22)(q34;q11),ins(11;18)(p15;q21q23) were the novel karyotypes observed in our CML-AP patients. The frequency of incidence *r*(10) was very rare in the myeloid neoplasm and was associated with bad prognosis in most of the reported cases. This was mainly due to permanent loss of distal genetic material and rearrangements of break point regions, resulting in fusion gene formation and ultimately contributed to the genomic instability of the cells (48). Likewise, the patient with *r*(10) in our study displayed a poor cytogenetic response which expired within 5 months of treatment with IM. In addition, cases with ins(11;18)(p15;q21q23) did not respond to IM therapy and eventually after 8 months the patient expired. 11p15/*NUP98* gene rearrangement was common in AML, MDS and T-ALL, but rare in CML and well-documented by their aggressive behavior and poor treatment outcome (49, 50).

In conclusion, the present study unravels the fact that ACAs are frequent in CML patients. But their frequency of incidence and distribution varies between different clinical stages of CML. This study identified that the genome of advanced phase patients were highly unstable and this environment of genomic instability is responsible for the high occurrence of non-random chromosomal anomalies or cytogenetic clonal evolution and genomic loss of sequences from both the derivative chromosome 9 and 22. Treatment response analysis revealed that compared to initial phases, ACAs were associated with an adverse prognostic effect during the progressive or advanced stages of CML. The synergic activity of all these aberrant cellular processes markedly influences the aggressiveness of disease progression in CML. Furthermore, the current study also warrants the need for performing molecular cytogenetic techniques like metaphase FISH and SKY analysis along with conventional cytogenetic analysis in CML cases to unveil the spectrum of chromosomal aberrations, especially in the advanced phases of the disease.

## Materials and Methods

### Patients

A total of 489 Ph chromosome/*BCR-ABL1* fusion gene positive CML patients, who attended the Medical Oncology out-patient clinics of Regional Cancer Center (RCC), Kerala, India during the period from January 2013 to January 2016, formed the study subjects. Both *de novo* and Ph/*BCR-ABL1* fusion gene positive CML patients, who were undergoing targeted drug therapy with IM for more than 1 year, were selected for the study. Thus, study subjects were categorized into four main clinical stages of CML; (i). *de novo* CML-CP patients, 313/489 (64%); (ii). CML-AP patients, 38/489 (7.77%); (iii). CML-BC patients, 60/489 (12.27%); and (iv). IM resistant CML-CP patients, 78/489 (15.95%) [The current study included both patients with primary and secondary resistance to IM]. All patients with a single copy of the Ph chromosome/ *BCR-ABL1*fusion gene were treated with IM at a dosage of about 400 mg daily; however, patients possessing multiple copies of the Ph chromosomes/ *BCR-ABL1*fusion genes were treated with 600–800 mg IM/day. Written informed consent for research purposes was obtained from all patients in accordance with the Declaration of Helsinki protocol. The study was approved by the Institutional Review Board and Human Ethics Committee of RCC (HEC #6/2010).

### Conventional Cytogenetic Analysis

Bone Marrow (BM) aspirate gathered from the patients was used for conventional cytogenetic analysis. BM cells were then cultured, harvested, and underwent chromosome analysis using GTG banding, performed according to a previously described procedure (51). G-banded karyotypes were constructed in each patient at the resolution of about 450 band levels and analyzed for chromosomal aberrations using cytogenetic software (ASI, Migdal Ha'Emek, Israel and Cytovision, USA). Karyotypes were described according to the International System for Human Cytogenetic Nomenclature (ISCN, 2016) guidelines.

### Molecular Cytogenetic Analysis

#### Fluorescence *in situ* Hybridization (FISH) Analysis

The FISH analysis, using LSI *BCR-ABL1* dual-color dual-fusion translocation probe (Abbott Molecular/Vysis, Des Plaines, IL, USA), was performed in all study subjects according to the manufacturer's instructions, to confirm the presence of the *BCR-ABL1* fusion gene (52). In addition, interphase and metaphase FISH analysis was performed on selected cases to confirm the chromosomal anomalies identified by cytogenetic analysis. For that, a total of 20 metaphase spreads and 200 interphase nuclei were analyzed using a fluorescence microscope (Olympus BX53, Tokyo, Japan). The list of LSI Vysis probes (Abbott Molecular, Des Plaines, IL, USA) used in the present study, other than *BCR-ABL1*, includes *RUNX1-RUNX1T1* and *CBFB-MYH11*. Apart from this, Chromosome X and Y Satellite Enumeration Probes and Whole Chromosome (WC) 2 DNA Probes (Kreatech Biotechnology B.V, 1032 LG Amsterdam, Netherlands) were also used in the study.

### Spectral Karyotyping (SKY) Analysis

The SKY analysis, which aids in the differential fluorescence of all 23 pairs of human chromosomes, was performed in selected CML cases to confirm the presence of complex chromosomal rearrangements identified by conventional cytogenetic analysis. Human SKY reagent or SKY probe mixture (ASI, MigdalHa'Emek, Israel) was applied to a 22 × 22 mm region of the metaphase preparation on the slides and were allowed to hybridize at 37°C for 24–36 h with optimal humidity following the manufacturer's protocol. Metaphase spreads were captured using a fluorescence microscope (Olympus BX53, Tokyo, Japan) equipped with appropriate filter sets to discriminate between a maximum of 5 fluorochromes and the counterstain DAPI. The hybridization signals were acquired with a Spectra Cube SD200 spectral imaging system and spectral karyotypes were constructed (ASI, Migdal Ha'Emek, Israel).

### Treatment Response Analysis

The data on the hematological and cytogenetic analysis of study subjects were documented initially at the time of diagnosis and these parameters were further validated periodically at a 3 months interval for the 1st year, and at a 6 months interval for the next 2 years after the initiation of IM therapy. Hematological and Cytogenetic response analysis were calculated as per a previously described protocol (53).

The Complete Hematologic Remission (CHR) was defined as the complete absence of immature cells and the normalization of White Blood Cells (WBC) and platelet counts in Peripheral Blood in association with entire reversal of splenomegaly. Patients with CHR showed a WBC count <10 × 10^9^/L and platelet count <450 × 10^9^/L. CHR also unveiled the complete disappearance of peripheral blast, immature granulocytes such as promyelocytes or myelocytes and <5% peripheral basophils. Criteria for partial hematologic response (PHR) is similar to that of CHR, except that there could be a persistence of immature cells, or platelet count <50% of the pre-treatment count but >450 × 10^9^ cells/L, or persistent splenomegaly but >50% of the pretreatment extent.

Cytogenetic response (CyR) is characterized by the percentage reduction of Ph positive metaphase cells from the bone marrow. Patients whose BM showed the complete absence of Ph positive metaphase cells is considered as complete cytogenetic response or CCyR, whereas the presence of 1–35% of Ph positive metaphase cells in the BM is called a major cytogenetic response or MCyR. Partial cytogenetic response or PCyR is denoted by the presence of 36–65% of metaphase cells with Ph, and minor or minimal cytogenetic response (mCyR) are BM cells with 66–95% Ph positive metaphase cells. Patients whose BM revealed more than 95% of Ph positive metaphase cells were categorized as IM non-responders or patients with no cytogenetic response (NCyR). MCyR includes both PCyR and CCyR.

### Statistical Analysis

Statistical calculations were carried out using the SPSS software version 21. A chi-square test and Fisher's exact test were used to compare the rate of incidence of ACAs between the study groups. The Student's *t*-test and Mann Whitney *U*-test were performed to reveal the difference in the distribution of covariates such as Hb (gm/%), TC (cmm), Platelet (cmm), LDH (IU/L), PB Blast (%), and BM blast (%) with the cytogenetic data obtained. A *P* < 0.05 was considered as statistically significant.

## Author Contributions

HS designed the research study. RK collected patient samples, performed conventional and molecular cytogenetic analysis, and wrote the manuscript. NG contributed the patient sample and clinical data. KS and RS were involved in the sample collection, cytogenetic analysis, and critically reviewed the manuscript. KJ performed the statistical analysis. All authors critically revised and agreed to the final version of the manuscript.

### Conflict of Interest Statement

The authors declare that the research was conducted in the absence of any commercial or financial relationships that could be construed as a potential conflict of interest.
